# Supplementary material for: Role of hydrogen bonding in hysteresis observed in sorption-induced swelling of soft nanoporous polymers
Source: Nat Commun. 2018 Aug 29;9:3507. doi: 10.1038/s41467-018-05897-9 (PMC6115358; doi:10.1038/s41467-018-05897-9)
Supplement: Supplementary file 1 — Supplementary Information [file 41467_2018_5897_MOESM1_ESM.pdf]

Supplementary Information

Role of hydrogen bonding in hysteresis observed in sorption-induced swelling of soft nanoporous polymers

Chen et al.

Supplementary Method

As explained in our paper, state-of-the-art techniques such as Molecular Dynamics (MD) and Grand Canonical Monte Carlo (GCMC) do not allow describing coupled adsorption/swelling in porous materials. Indeed, while the former requires to work with a constant number of molecules, the latter only applies to systems having a constant volume. Following previous works, including the work by Ghoufi and Maurin for adsorption in Metal Organic Frameworks[1], we use a hybrid molecular simulation that consists of combining GCMC and MD to perform simulations in the so-called osmotic statistical ensemble $\mu\sigma T$ where μ is the chemical potential of water, σ is the stress applied to the cellulose material, and T the temperature. To do so, part of the Markov chain used in the Grand Canonical Monte Carlo simulations is replaced by an MD trajectory at constant number of water molecules N, constant stress σ and temperature T. More in details, this means that, in addition to conventional MC steps in the GCMC algorithm (insertions and deletions), MD timesteps *i.e.* at constant number of molecules (allowing for water molecule translations or rotations and cellulose local relaxation at constant external stress) are added as the MD technique is more efficient at converging towards local equilibrium. Moreover, by using MD simulations in the isostress and isothermal ensemble, the host (hydrated) cellulose material is allowed to swell or shrink. As shown in previous works, such a hybrid strategy succeeds in capturing coupled adsorption/swelling phenomena including adsorption-induced phase transitions in breathing materials such as Metal Organic Frameworks [1].

In practice, our hybrid molecular simulations were performed at T = 300 K using a Berendsen thermostat and an anisotropic external stress σ = 0 Pa (with the following relaxation times: τ_T = 0.1 ps and τ_s = 1.0 ps). Water molecules are described using the SPC/E water model with the SHAKE algorithm to maintain its internal structure rigid. The MD trajectory was integrated using the velocity Verlet integrator with a timestep equal to 1 fs. Hybrid MD/GCMC molecular simulations consisted of performing a large number of blocks where one block corresponds to 2,000 GCMC insertion/deletion attempts followed by 200 MD timesteps. In total, 10^5 blocks were first performed to equilibrate the system

followed by 2×10^4 additional blocks to accumulate statistics. In order to check the efficiency of the phase space sampling, we monitored the pressure and volume along the hybrid GCMC/NσT simulations. Supplementary Figure 1 shows the volume of the simulation box as a function of the number of time steps in the molecular dynamics simulation N_{MD}. We recall that, after every 200 MD timesteps, the simulation procedure also includes a GCMC segment that consists of 2,000 MC steps. The insert in Supplementary Figure 1 shows for two different chemical potentials μ how the volume fluctuates around its equilibrium value in the course of a block consisting of a MD segment and a GCMC segment (of course, the volume does not change during the GCMC segment as only the energy and number of molecules are allowed to change). Overall, the data in Supplementary Figure 1 show that volume sampling in such hybrid GCMC/NσT simulations is efficient and that the volume reaches its final equilibrium value provided simulations are long enough (typically, at high loadings, at least 10^7 MD timesteps are required but shorter simulations are needed for low loadings). We also show in Supplementary Figure 2 the stress monitored during a MD segment. As expected, owing to the system size, fluctuations over the stress are large but we checked that its average value is equal to the stress imposed in the simulations. We note that the stress variations seen in Supplementary Figure 2 are small compared to the stiffness of the material, ~10 GPa (as also reflected by the small volume variations in Supplementary Figure 1).

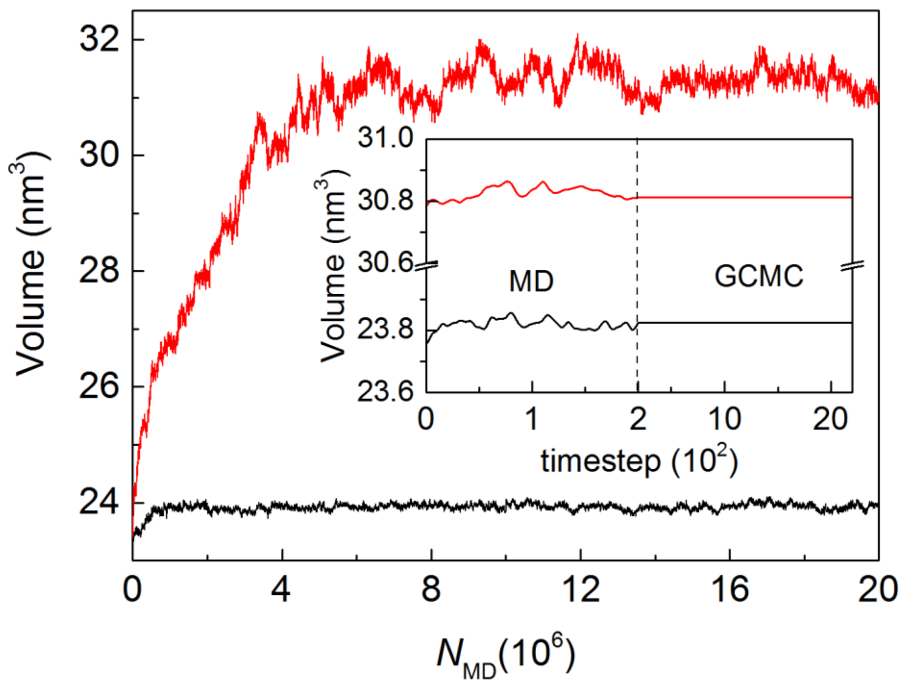

Supplementary Figure 1. Volume in nm^3 as a function of MD steps as obtained from hybrid GCMC/MD simulations. The data obtained for a relative humidity RH = 0.025 (black data) and RH = 1 (red data) are shown. The insert, which shows a zoom corresponding to the end of the hybrid simulation, illustrates how the volume fluctuates around its equilibrium value in the course of a block consisting of a MD segment and a GCMC segment (the volume does not change during the GCMC segment as only the energy and number of molecules are allowed to change).

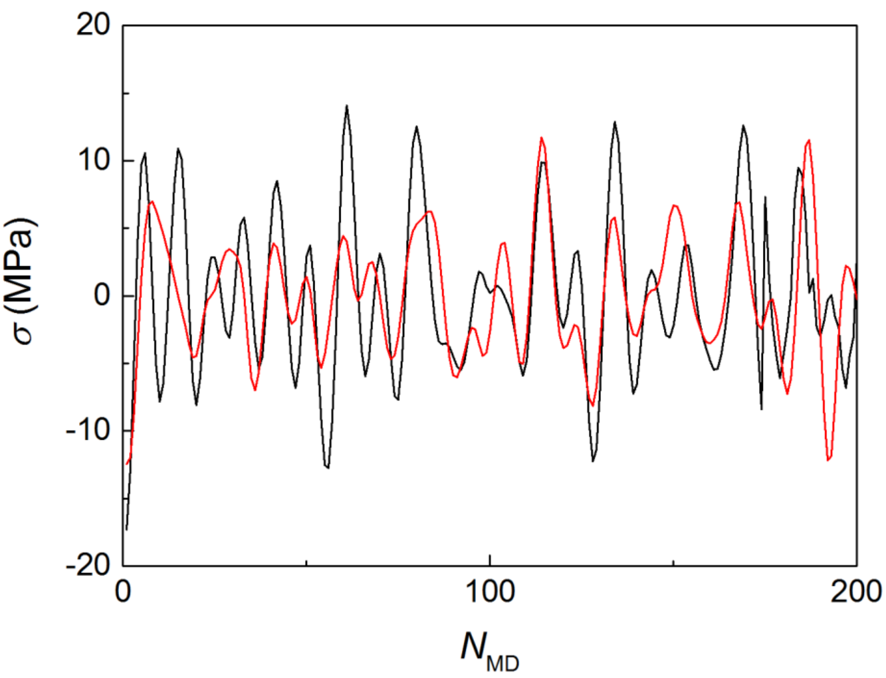

Supplementary Figure 2. Stress fluctuations in the course of a MD block performed in a hybrid GCMC/MD simulation. The data obtained for a relative humidity RH = 0.025 (black data) and RH = 1 (red data) are shown.

We also checked for two water chemical potentials that the number of water molecules converges to its final equilibrium value. Supplementary Figure 3 shows the number of water molecules N_{water} as a function of the number of GCMC steps N_{GCMC} (as explained above, such hybrid simulations also include MD segments which are not shown here for the sake of clarity since the number of molecules is constant in MD).

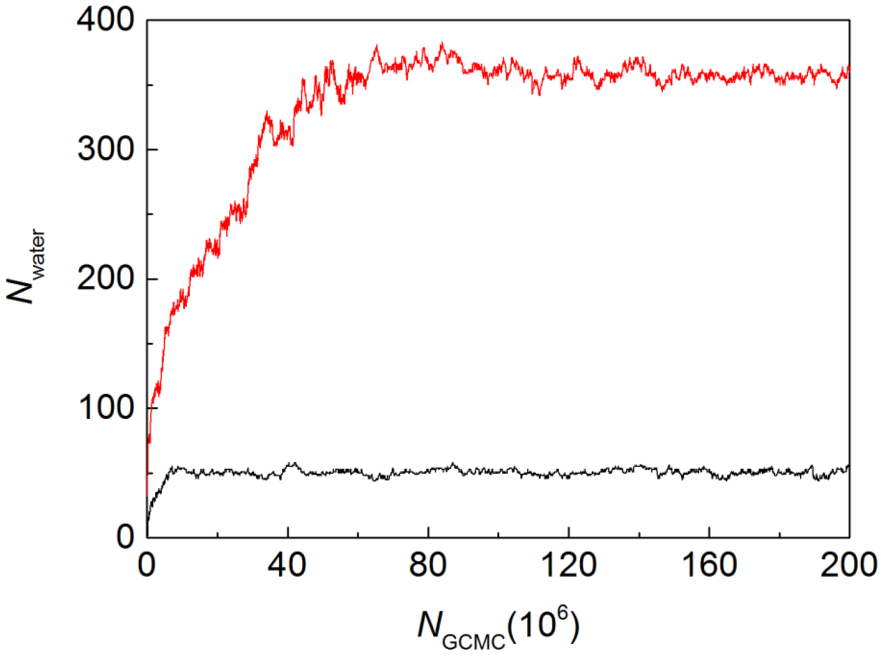

Supplementary Figure 3. Number of water molecules N_{water} as a function of GCMC steps as obtained from hybrid GCMC/MD simulations. The data obtained for a relative humidity RH = 0.025 (black data) and RH = 1 (red data) are shown (N_{water} in the course of the MD segments is not shown because it is not allowed to change in the NσT ensemble).

There is a non-negligible mismatch between the experimental saturating vapor pressure for water and its numerical counterpart when using the SPC/E water model. This raises the question whether adsorption isotherms should be compared when plotted as a function of reduced pressure P/P_0 or absolute pressure P. Considering the fundamental definition of adsorption phenomena (in terms of Gibbs adsorption equation or Polanyi theory for instance), the thermodynamic parameter that governs the adsorbed amount is the chemical potential difference $\Delta\mu = \mu - \mu_0$ with respect to the chemical potential at saturation μ_0. Indeed, at the saturation chemical potential i.e. at the bulk gas/liquid coexistence, adsorption from a relative humidity RH = 1 ($P = P_0$) is maximum (any adsorption above μ_0 corresponds to intrusion and is therefore only relevant to hydrophobic materials). As a result, when comparing adsorption isotherms for different fluids, the natural choice is to use the chemical potential difference $\Delta\mu = \mu - \mu_0$. This is relevant when comparing different

fluids but also different models for the same fluid when using molecular simulation (or as in the present paper when comparing experimental and simulation data). Considering that water vapor at room temperature behaves as an ideal gas, we used the following relationship RH = $P/P_0 = \exp[\Delta\mu/k_B T]$.

Supplementary Discussion

PCFF is a force field parameterized against a broad range of experimental observables for organic compounds. It has been applied to modelling cellulose-based materials by many researchers who have found that it captures quantitatively or semi-quantitatively most physical properties. More in details, for instance, Chen et al.[2] built an amorphous cellulose model and showed that the final density obtained is consistent with its experimental counterpart (1.39 g cm^{-3} *versus* 1.48 g cm^{-3}). Similarly, in the present work, we found that the final density of our models (ranging from 1.39 to 1.41 g cm^{-3}) is consistent with typical experimental densities for cellulose (see Supplementary Table 1). As far as mechanical properties are concerned, Tanaka and Iwata [3] used molecular simulation with the same force field to assess Young's modulus of cellulose crystals; these authors found values in the range 124-155 GPa that are in good agreement with the experimental value (~138 GPa)[4] (no experimental mechanical data are available for amorphous cellulose). As for adsorption properties, in their molecular simulation of adsorption onto cellulose, Da Silva Perez et al.[5] found that the heat of adsorption for a large variety of aromatic compounds is consistent with their experimental counterpart (84% of the adsorbate-cellulose couples displayed differences < 20% between the measured and predicted heats of adsorption). Xu and Chen[6] also found that PCFF predicts formaldehyde diffusion in cellulose with a temperature dependence of the self-diffusion coefficient in good agreement with the experimental data. Finally, in the context of the present work on water adsorption/desorption in cellulose, we emphasize that PCFF leads to cellulose/water hydrogen bonds with a typical energy (5.4 kcal mol^{-1}, see discussion in our manuscript) that is consistent with the conformational analysis made by Pizzi et al.[7,8]; these authors estimated theoretically that the sorption energy is around 5.5 kcal mol^{-1} for cellulose I crystals and 6.5 kcal mol^{-1} for paracrystalline (amorphous) cellulose. Overall, the discussion above shows that the forcefield used in the

present work provides a reasonable, at least semi-quantitative, description of cellulose (including its density, mechanical, and adsorption properties).

Supplementary Table 1. Configurational information on the three cellulose samples prepared and comparison with previous experiment/simulation.

Sample Number	1	2	3	Exp. [9]	Sim. [9]
Number of chains	5	4	3	-	1
Degree of polymerization	10	16	20	-	20
Total number of atoms	2110	2696	2526	-	842
Density (g cm^{-3})	1.395	1.392	1.409	1.48	1.385

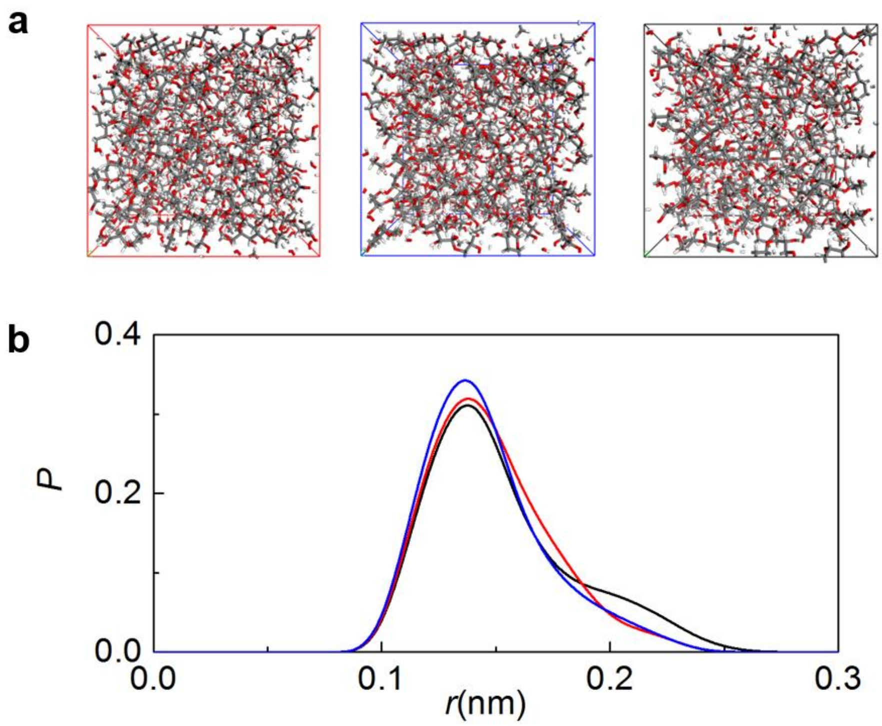

Supplementary Figure 4. (a) Molecular configurations of the three samples after relaxation. The sticks are the bonds between the C, O, and H atoms in cellulose with C, O, and H being grey, red and white respectively. (b) Pore size distribution for the three dry cellulose samples. The red, blue, and black lines are for samples 1, 2, and 3 respectively. For all samples, the pore sizes are less than 0.25 nm.

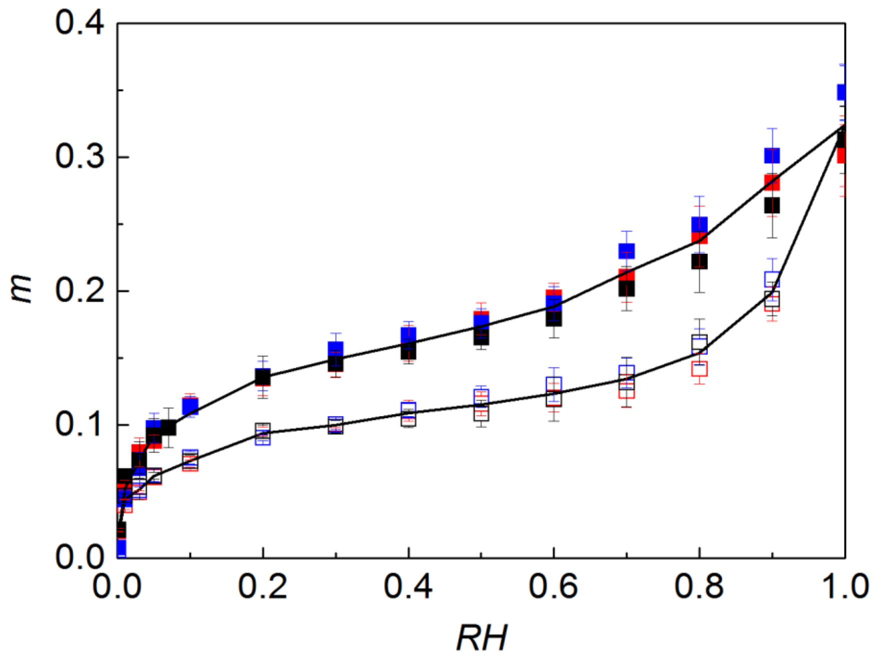

Supplementary Figure 5. Water adsorption/desorption isotherms at room temperature for the three cellulose samples. The red, blue, and black squares are for samples 1, 2, and 3 respectively. The open and closed symbols are the adsorption and desorption data, respectively. The solid lines represent the adsorption and desorption data averaged over the three samples. The error bar is defined as the standard deviation (s.d.) of *m* during the last 2×10^4 simulation blocks.

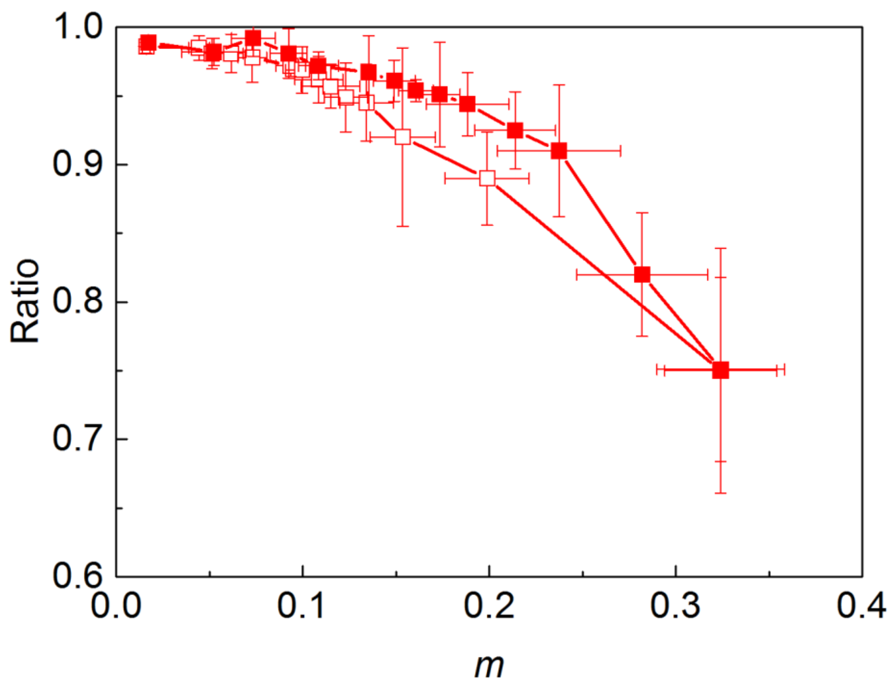

Supplementary Figure 6. Fraction of water molecules hydrogen-bonded to the cellulose chains. Open and closed symbols correspond to adsorption and desorption, respectively. The error bar is defined as the standard deviation (s.d.) of the 3 samples.

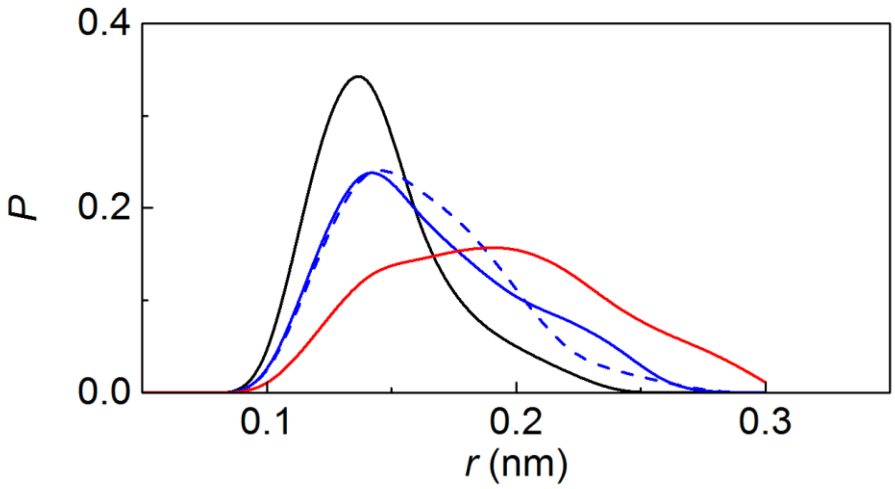

Supplementary Figure 7. Pore size distribution of the cellulose samples at three different saturation states indicated by the black, red and blue dashed line in (a) and (b): the dry state m = 0 (black solid line), state close to RH = 1 (red solid line) and intermediate state m =

0.151 upon adsorption (blue solid line), and intermediate state m = 0.154 upon desorption (blue dashed line).

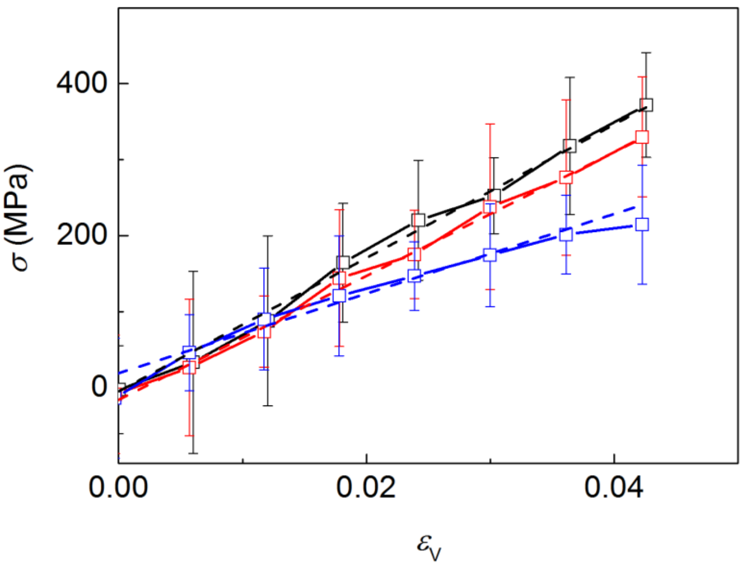

Supplementary Figure 8. Typical stress-strain curves determined from a triaxial tensile test using MD simulation at constant strain rate and temperature during adsorption of sample 1. The black, red and blue solid lines correspond to m=0, m=0.15 and m=0.32 respectively. The error bar is defined as the standard deviation (s.d.) of the 3 samples.

Supplementary Reference

1. Ghoufi, A. & Maurin, G. Hybrid monte carlo simulations combined with a phase mixture model to predict the structural transitions of a porous Metal-organic framework material upon adsorption of guest molecules. *J. Phys. Chem. C* **114,** 6496–6502 (2010).

2. Chen, W., Lickfield, G. C. & Yang, C. Q. Molecular modeling of cellulose in amorphous state. Part I: Model building and plastic deformation study. *Polymer (Guildf).* **45,** 1063–1071 (2004).

3. Tanaka, F. & Iwata, T. Estimation of the elastic modulus of cellulose crystal by molecular mechanics simulation. *Cellulose* **13,** 509–517 (2006).

4. Nishino, T., Takano, K. & Nakamae, K. Elastic modulus of the crystalline regions of cellulose polymorphs. *J. Polym. Sci. Part B Polym. Phys.* **33,** 1647–1651 (1995).

5. Da Silva Perez, D., Ruggiero, R., Morais, L. C., Machado, A. E. H. & Mazeau, K. Theoretical and experimental studies on the adsorption of aromatic compounds onto cellulose. *Langmuir* **20,** 3151–3158 (2004).

6. Xu, B. & Chen, Z. Formaldehyde diffusion within crystalline and amorphous cellulose at different temperatures and electric fields: A molecular dynamics study. *Indoor Built Environ.* **0,** 1–11 (2017).

7. Pizzi, A., Bariska, M. & Eaton, N. J. Theoretical water sorption energies by conformational analysis-Part 1: Crystalline cellulose I. *Wood Sci. Technol.* **21,** 235–248 (1987).

8. Pizzi, A., Bariska, M. & Eaton, N. J. Theoretical water sorption energies by conformational analysis-Part 2: Amorphous cellulose and the sorption isotherm. *Wood Sci. Technol.* **21,** 317–327 (1987).

9. Mihranyan, A., Llagostera, A. P., Karmhag, R., Strømme, M. & Ek, R. Moisture sorption by cellulose powders of varying crystallinity. *Int. J. Pharm.* **269,** 433–442 (2004).
